# Ensemble methods for stochastic networks with special reference to the biological clock of *Neurospora crassa*

**DOI:** 10.1371/journal.pone.0196435

**Published:** 2018-05-16

**Authors:** C. Caranica, A. Al-Omari, Z. Deng, J. Griffith, R. Nilsen, L. Mao, J. Arnold, H.-B. Schüttler

**Affiliations:** 1 Department of Statistics, University of Georgia, Athens, Georgia; 2 Department of Biomedical Systems and Informatics Engineering, Yarmouk University, Irbid, Jordan; 3 School of Electrical and Computer Engineering, College of Engineering, University of Georgia, Athens, Georgia; 4 Genetics Department, University of Georgia, Athens, Georgia; 5 College of Agricultural and Environmental Sciences, University of Georgia, Athens, Georgia; 6 Department of Physics and Astronomy, University of Georgia, Athens, Georgia; University of Edinburgh, UNITED KINGDOM

## Abstract

A major challenge in systems biology is to infer the parameters of regulatory networks that operate in a noisy environment, such as in a single cell. In a stochastic regime it is hard to distinguish noise from the real signal and to infer the noise contribution to the dynamical behavior. When the genetic network displays oscillatory dynamics, it is even harder to infer the parameters that produce the oscillations. To address this issue we introduce a new estimation method built on a combination of stochastic simulations, mass action kinetics and ensemble network simulations in which we match the average periodogram and phase of the model to that of the data. The method is relatively fast (compared to Metropolis-Hastings Monte Carlo Methods), easy to parallelize, applicable to large oscillatory networks and large (~2000 cells) single cell expression data sets, and it quantifies the noise impact on the observed dynamics. Standard errors of estimated rate coefficients are typically two orders of magnitude smaller than the mean from single cell experiments with on the order of ~1000 cells. We also provide a method to assess the goodness of fit of the stochastic network using the Hilbert phase of single cells. An analysis of phase departures from the null model with no communication between cells is consistent with a hypothesis of Stochastic Resonance describing single cell oscillators. Stochastic Resonance provides a physical mechanism whereby intracellular noise plays a positive role in establishing oscillatory behavior, but may require model parameters, such as rate coefficients, that differ substantially from those extracted at the macroscopic level from measurements on populations of millions of communicating, synchronized cells.

## Introduction

Gene regulation is an intrinsically stochastic process[1–3]. The low copy numbers of some molecules, such as genes, involved in gene regulation lead to a noisy time series of numbers of molecular species in a gene regulatory network within a single cell. This randomness can produce different phenotypes for genetically identical organisms[4, 5]and for a single transcription factor[3]. This randomness can also produce coordinated regulation of target genes[6], and for a combination of 2 or more transcription factors, combinatorial regulation by changes in relative pulse timing between transcription factors[7], and have a role in the evolution of genetic networks[8]. To measure this stochasticity and to extract information about the regulatory network from the numbers of molecular species over time has become a major challenge in systems biology[9, 10]. Recent progress in addressing this task has been due mainly to advances in high-throughput single-cell measurement techniques for measuring gene expression, yielding large datasets on gene expression in single cells and the development of computational models used to explain these data[11–15].

Computational models should be able to capture the main features of the experimental data, such as the histories of molecular species in a cell, and provide new insights about the biological process operating in single cells[16, 17]. To build such a model, a critical step is to quantify the many unknown parameters that characterize the behavior of a single cell[18]. For genetic networks describing single cells these parameters include, for example, reaction rate coefficients, initial molecular numbers, mRNA/DNA ratios, and Hill coefficients. These quantities are difficult to measure directly on single cells. Usually only a few of those predicted by the model are available from experiments, such as the levels of a few proteins or mRNAs, observed through their fluorescence[14, 19].

In the context of gene regulation, we need to simulate the behavior of whole gene networks in single cells to fit these models. One of the earliest methods to simulate stochastic gene networks was developed by Gillespie[20]. It allows exact simulation of stochastic biochemical networks, in principle for any duration of time and network size. By measuring the trajectories of many cells, we can find desired statistical summaries of the period, phase, and amplitude for the time series of molecular numbers in a cell. By comparing these with analogous summaries generated by a stochastic model, we can infer parameters of the underlying stochastic process. The only drawback of Gillespie’s method is that it can take a long time to run, and generating summary statistics with a high degree of accuracy can be computationally prohibitive. Approximate stochastic simulation methods can be used to speed up the computations, but introduce additional errors that are difficult to account for in the model fitting process.

The *τ*-leaping methods[21] is such an approximation to the exact Gillespie algorithm. Instead of simulating a succession of reactions one at a time, a Poisson distribution is used to approximate the number of times each reaction is occurring in that time interval. This can decrease the simulation time significantly if certain conditions are met. But the Poisson approximation introduces additional error, and supplementary computations are then needed to verify that the approximation is accurate.

Maximum-likelihood methods have also been used for fitting stochastic networks[22]. These methods select those parameter values that maximize the likelihood that the model generates the observed data. The main difference among these approaches is the way the maximum-likelihood estimator is calculated. Some of the methods use Markov Chain Monte Carlo Methods[23, 24], to provide a direct solution of the stochastic model’s maximum likelihood estimator or linear approximations thereof[25]. Although these existing methods work well for small networks, they become too cumbersome for larger networks. Many of them produce only point estimates of the network parameters, which cannot capture the behavior of the system due to the noise in these point estimates. Other approximate methods of stochastic network identification have been recently proposed using either moment-closure or volume expansion methods to approximate the chemical master equation describing the stochastic network to simplify the fitting problem[26].

Ensemble methods solve this problem. Based on a Bayesian posterior distribution or likelihood function, they produce large samples of parameter values consistent with observed data that can then be model-averaged to capture the system behavior[27]. Consequently, they can produce confidence intervals of the model parameters[28]. More recently, these methods have evolved into Approximate Bayesian Computation (ABC) and have been used successfully in other biological contexts[27, 29]. For large networks, ensemble-based parameter inference methods employ Markov Chain Monte Carlo (MCMC) simulations techniques to draw samples from the high-dimensional model parameter spaces[27, 30, 31]. These MCMC simulations are highly CPU time consumptive and one of the challenges is then to find efficient computational approaches to make these simulations feasible within reasonable computation time limits.

In modeling stochastic molecular time series data, it is important to notice that the individual random trajectories of molecule numbers cannot, in general, be compared directly to individual observed single-cell fluorescence time series. The stochastic variability of the individual trajectories in both model and experiment preclude a meaningful comparison. In the context of potentially oscillatory data, as expected in the biological clock system, it is also not useful to compare the average of model molecular time series to the average over the observed single fluorescent data from all cells. Both model and observed trajectories are typically randomly phase-shifted relative to each other, and averaging them therefore tends to cancels out the oscillatory part of the signal. Consequently, it is important to design meaningful summary statistics which preserve the information about the oscillatory signal, including oscillation periods, phases and amplitudes, when averaged over all cells and model trajectories, respectively[32–34].

One aim of this paper is to provide a fast, computationally feasible method for parameter inference in a stochastic oscillatory biochemical network and show its successful application to understanding one of the best studied biological clocks at the molecular level[35]. The method proposed uses different MCMC methods, such as Metropolis-Hastings and parallel tempering, to fit the average periodogram[36], also known as the power spectrum, of the model to the average periodogram of the data, where the average is taken over the periodograms of individual cell fluorescent trajectories. The periodogram is a summary statistic which preserves two of the relevant features of an oscillatory process, its amplitude and period. We use deterministic mass action kinetics to initialize the Markov chains with parameter values that produce small chi-squared values relatively fast on General Purpose Graphics Processor Units (GPGPUs)[37]. Thus, we can rapidly obtain model parameter sets that capture the important periods and amplitudes in the data. These models can be further used to match the observed phases to test the adequacy of the models.

A second aim of the paper is to explore whether the oscillations in such a network, might actually be caused by or reinforced by the molecular noise in the cell through a Stochastic Resonance-like phenomenon[38–40]. Stochastic Resonance is a theory that arose in physics[38] to explain the behavior of physical oscillators. Under the Stochastic Resonance hypothesis the stochastic intracellular noise is assumed to have a positive role in generating periodic behavior provided this noise is not too little or too large in magnitude. The key to the Stochastic Resonance hypothesis is that the presence of oscillations has a nonlinear relation with the level of stochastic intracellular noise.

**Fig 1:**
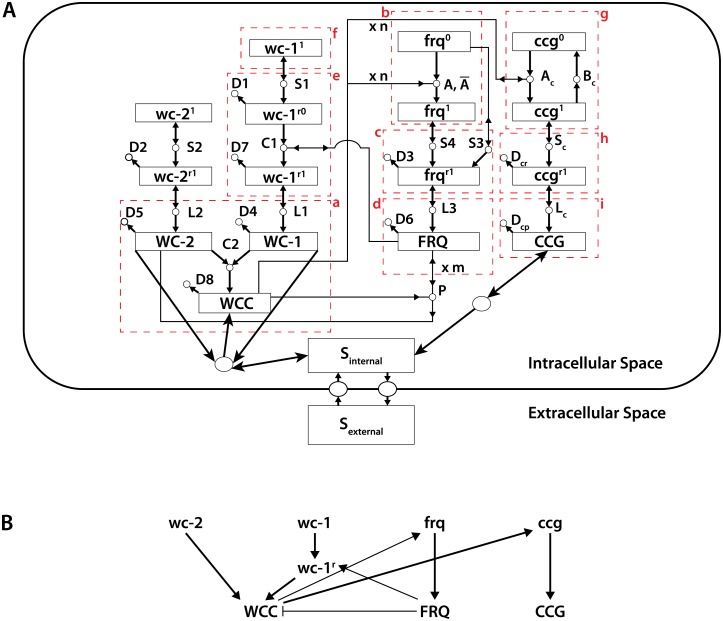
(A) The stochastic clock network of *Neurospora crassa* in a single cell. From[13, 28]. While an exometabolite S_external_ is being produced, there are no other cells in a droplet to sense this signal for the data described in Materials and Methods. The boxes outlined (in red) with dashed lines and labeled a through i in red, define regions of the network between which there is little or no net flow of molecules. These boxes are utilized to define scale factors for converting the concentrations of molecules in the deterministic network to molecular numbers in the stochastic network while preserving the network dynamics as described in the Materials and Methods. A few reactions are outside the boxes because in fitting the model WC-2 was assumed to be constant. The reaction of WCC to reaction A produces a small flow into a box, but to good approximation the role of WCC in the A reaction can be viewed as catalytic if the number of molecules of WCC is in the hundreds. (B) Cartoon of the stochastic network highlighting important features in panel A. Arrows are used to indicate a positive effect. A line with a bar (-|) is used to indicate a negative effect.

It is therefore important in explaining oscillations in a stochastic network to infer the impact that noise has on cellular mechanisms and to quantify how these mechanisms respond to different noise levels. Our model and methods to fit a stochastic network (Fig 1) were developed with these purposes in mind.

## Model

Our models simulate a well-stirred biochemical system with N molecular species {*S*_1_, *S*_2_, …, *S*_*N*_} having discrete-valued molecular numbers given by *X* = {*X*_1_, *X*_2_, …, *X*_*N*_}. These molecular numbers change in time through the firing of M reactions {*R*_1_, *R*_1_, …, *R*_*M*_}. The state of the system at a time *t* is given by the random vector *X*(*t*) = {*X*_1_(*t*), *X*_2_(*t*), …, *X*_*N*_(*t*)} with *X*_*i*_(*t*) being the number of molecules of species *S*_*i*_ at time *t*, *i* = 1, …, *N*.

Knowing the state of the system at time *t*, *X*(*t*) = *x*, we assign to each reaction *R*_*j*_ a propensity function *a*_*j*_(*x*) whose product with an infinitesimal time increment *dt* determines the probability that reaction *R*_*j*_ fires in the next infinitesimal time interval [*t*, *t* + *dt*). These propensity functions *a*_*j*_(*x*), *j* = 1, …, *M*, are defined based on mass action kinetics, *a*_*j*_(*x*) = *k*_*j*_*b*_*j*_(*x*) where *k*_*j*_ is a kinetic constant specific to reaction *R*_*j*_ and *b*_*j*_(*x*) counts the number of ways reaction *R*_*j*_ can occur given state *X*. For instance, for mono-molecular, homo-bimolecular and hetero-bimolecular reactions *b*(*x*) takes the form *x*_1_, *x*_1_ (*x*_1_ − 1)/2 and *x*_1_*x*_2_, respectively. The sum of all propensities is denoted by *a*_0_(*x*).

The parameters we need to infer are initial molecular numbers and the kinetic constants, Θ = {*X*_1_(0), *X*_2_(0), …, *X*_*N*_(0), *k*_1_, …, *k*_*M*_}. Gillespie showed that from knowing these parameters we can build an exact sample trajectory of the network to find the molecular numbers at any later time. Thus, a parameter set Θ gives a model of the network’s dynamics.

Our study is focused on a well-studied oscillatory network, the clock network of *Neurospora crassa*[28]. This network is presented in Fig 1. The species with superscript r denotes an mRNA; the ones in capital letters are proteins. The rest are genes. As shown in Yu *et al*. [28], we can consider the protein WC-2 to be constant, so we can ignore the species *wc-2*^*1*^, *wc-2*^*r1*^ and the reactions in which they are involved. Also, *wc-1*^*1*^ is constant. Thus, we reduce the network to 12 molecular species and 22 reactions, which makes our parameter space 34-dimensional. Earlier work[28] has shown this is a good approximation. This particular model is in one of two classes of negative feedback models for clocks in different organisms termed a Hill-type transcriptional repression model[41, 42].

Some essential features of the model are captured in the cartoon (Fig 1B). The genes *white collar-1* (*wc-1*) and *white collar-2* (*wc-2*) produce a heterodimer WCC = WC-1/WC-2, which activates the oscillator gene *frequency* (*frq*) and a *clock-controlled gene* (*ccg*). The FRQ oscillator protein in turn provides a negative feedback loop to deactivate WCC. The genes *wc-1* and *wc-2* encode the positive elements in the clock mechanism, and *frq* encodes a negative element[28]. The FRQ protein also appears to have a role in stabilizing the *wc-1* mRNA (*wc-1*^*r*^)[28].

## Materials and methods

### Single cell data of *N*. *crassa*

Two single cell data sets were used[13]. One data set has 868 single cells; the second one as a replicate has 1591 single cells. These two data sets were generated through time-dependent oscillatory fluorescent measurement on single *N*. *crassa* cells encapsulated in aqueous droplets of ~100 um in diameter and physically separated from each other as a result. The measurements were through the use of a fluorescent recorder (mCherry) linked to a promoter on a *clock controlled gene-2* (*ccg-2*)[43]. We obtained one data set including the time series of 868 single cells over ten days, and another data set including the time series of 1,591 single cells over ten days. The second data set is attached as a supplementary excel file. An improved cell-tracking method was used to bring the data set to 1,644 cells from 1,591 cells as originally described[13]. In the supplemental spread sheet each column is a different cell, and each row, a different time point. There are 563 time points per cell taken from time 0 to time 261.5 hours every half hour. Each single cell time series is Rhodamine B normalized, detrended, and bias-corrected[13]. Only 61^st^ to 540th time points were used in the analysis to allow each oscillator the opportunity to reach a stable limit cycle and to maintain cell viability at the end of the series.

### Rescaling from deterministic model units to stochastic molecular number units

In previous work[28] our clock network was studied in a deterministic framework. An ensemble of oscillating network models quantitatively consistent with available RNA and protein profiling data was found. If we wish to use concentration results from these deterministic models as inputs in a stochastic framework, then we need to rescale the initial molecular concentrations in the deterministic model to molecular numbers in the stochastic model, that is non-negative integers counting molecules in Fig 1, while preserving the deterministic dynamics[44]. This conversion reduces to a change in measurement units for each species such that the total gene concentration of a species in the new molecular number units is 1 in a single cell, and the time-averaged mRNA and protein concentrations in molecular number units are equal to the observed RNA:DNA and protein:DNA ratio, respectively. The RNA:DNA and protein:DNA ratios were determined experimentally, as described below, and summarized in Table 1. The RNA:DNA and protein:DNA ratios used below were 128.7 and 412, respectively, averages from Table 1.

**Table 1:** The protein/RNA/DNA ratios used for specifying the scale parameters in a stochastic network were measured and reported below. For each of 13 evenly spaced time points over 48 hours, a sample was taken. A commercial kit was used to simultaneously extract DNA, RNA, and protein from each sample. The amounts of DNA, RNA, and Protein were then measured. To convert these measurements to nanomoles the average molecular weight of a protein and RNA was computed. The average molecular weight of an amino acid is 128.0452. Hence the average molecular weight of a protein in *N*. *crassa* was taken as 481*128.0452. The average molecular weight of an RNA was taken as 1673*(propA*329.2+propC*305.2+propG*345.2+propU*306.2)+159, where the proportion of A (propA) etc was taken from[46].

Time	DNA ng/*μ*l	RNAng/*μ*l	Protein ng/*μ*l	DNA (nanomoles)	RNA(nanomoles)	Prot (nanomoles)	RNA:DNA	Prot:DNA	Prot:RNA
0	8.8	1063	422	1.70495E-05	0.001974891	0.006851792	115.8330089	401.8772813	3.469453874
4	6.5	1511	248	1.25934E-05	0.002807206	0.004026645	222.9116944	319.7437035	1.434396273
8	7.5	1036	292	1.45308E-05	0.001924729	0.00474105	132.4586234	326.2761018	2.463230353
12	7.6	1062	550	1.47245E-05	0.001973033	0.00893006	133.9962578	606.4748197	4.526057888
16	15	1092	347	2.90616E-05	0.002028768	0.005634056	69.80927451	193.8661084	2.777082412
20	4.4	1051	261	8.52473E-06	0.001952596	0.00423772	229.0507852	497.1088646	2.170299762
24	7.2	1084	683	1.39496E-05	0.002013905	0.011089511	144.3705234	794.9720944	5.506470958
28	18.2	1216	220	3.52614E-05	0.002259141	0.003572024	64.06840795	101.3012886	1.581142591
32	5.9	1170	387	1.14309E-05	0.00217368	0.006283515	190.1584354	549.6960677	2.890726707
36	13.5	1311	343	2.61554E-05	0.002435636	0.00556911	93.12165128	212.9237118	2.286511341
40	14	1161	369	2.71241E-05	0.002156959	0.005991259	79.5217501	220.8828551	2.777640769
44	13.8	1159	697	2.67367E-05	0.002153244	0.011316822	80.53526552	423.2698834	5.255708547
48	7.4	904	626	1.4337E-05	0.001679493	0.010164032	117.1435702	708.9347917	6.051845528
Ave.							128.7	412	

Following an established notation[28], the 12 species concentrations [wc-1^r0^], [wc-1^r1^], [WC-1], [WCC], [frq^0^], [frq^1^], [frq^r1^], [FRQ], [ccg^0^], [ccg^1^], [ccg^r1^], and [CCG] are abbreviated here to *u*_*r*0_, *u*_*r*1_, *u*_*p*_, *w*, *f*_0_, *f*_1_, *f*_*r*_, *f*_*p*_, *g*_0_, *g*_1_, *g*_*r*_, *g*_*p*_, respectively, with constant total gene concentrations *f*_*G*_ = *f*_0_ + *f*_1_ and *g*_*G*_ = *g*_0_ + *g*_1_. To convert the parameters of the network from deterministic model units to molecular number units of counts of molecules in a cell we do the following:

1 divide the network into separate components or “boxes” in such a way that two different boxes will be connected only through catalytic reactions (Fig 1). So, there will be no net flow of molecules between different boxes. Thus, the scales of measurement units between boxes can be varied independently without changing network dynamics. We obtain 9 boxes denoted by letters from *a* to *i*. They are, a: *w*, *v*_*p*_, *u*_*p*_, b: *f*_0_, *f*_1_, c: *f*_*r*_, d: *f*_*p*_, e: *u*_*r*0_, *u*_*r*1_, f: *v*_*r*_, g: *g*_0_, *g*_1_, h: *g*_*r*_ and i: *g*_*p*_.
2 If we denote the model units by *mu* and real molecular number units by *ru*, then knowing the value of a concentration parameter expressed in *mu*, we need to convert it to a value that uses *ru*. We just need to find the ratio $\frac{mu}{ru}$. Each box will have its own $\frac{mu}{ru}$ ratio. For an arbitrary box *z*, we denote by ${\left(\frac{mu}{ru}\right)}_{z}$ its conversion ratio.

For the box containing a gene, like box *b* with species *f*_0_, *f*_1_, we have the values $\frac{f_{0}}{mu},\frac{f_{1}}{mu}$ = (.356365, .0824576) from the deterministic model in Table 1 (column 2), so we know $f_{G,mu}\;=\;\frac{f_{0}+f_{1}}{mu}\;=\;0.4388226$.

We also know $\frac{f_{0}+f_{1}}{ru}\;=\;1$, because there is just one *frq* gene in the cell.

Then ${\left(\frac{mu}{ru}\right)}_{b}\;=\;\frac{1}{f_{G,mu}}\;=\;2.278825$.

When applying this conversion factor, a gene is converted to the nearest whole gene so there isn’t a fractional gene.

For boxes with an mRNA we take the average value of an mRNA concentration parameter over a simulated trajectory obtained using the deterministic model and compare it to the RNA:DNA ratio. If the simulated trajectory contains a transient signal, we discard the corresponding part of the trajectory. Since the deterministic models display sustained oscillations, we want our mRNA deterministic values to come from the purely oscillatory part of the solution (not the transient part).

Thus, for box *c* we find $f_{r,mu}\;=\;\frac{\overset{-}{f_{r}}}{mu}\;=\;\frac{1}{t_{1}-t_{0}}\int_{t_{0}}^{t_{1}}\frac{f_{r}(t)}{mu}dt$, where *fr*(*t*) is the value of *fr* at time *t* in the deterministic simulation of the network between times *t*_0_ and *t*_1_. In the time interval [*t*_0_, *t*_1_) the deterministic trajectory traced 10 complete cycles.

Also, $\frac{\overset{-}{f_{r}}}{ru}\;=\;R_{RNA:gene}$ is the RNA:DNA ratio for *frq* species, namely 128.7. This ratio is experimentally determined from Table 1.

Then, ${\left(\frac{mu}{ru}\right)}_{c}\;=\;\frac{R_{RNA:gene}}{f_{r,mu}}=128.7/0.02319352=5548.963$.

Similarly, for boxes with a protein we will use Protein:DNA ratio of 412 of the corresponding species. All box ratios can be found in this way. **For box d,**

$${\left(\frac{mu}{ru}\right)}_{d}\;=\;\frac{R_{Prot:DNA}}{f_{p,mu}}\;=\;\frac{412.1}{0.46295}\;=\;890.1612$$

The ratios RNA:DNA and Protein:DNA were found experimentally from Table 1.

Then, to convert a molecular concentration given by a deterministic model to a molecular number we just multiply the molecular number by the conversion ratio of the box to which the species belongs. To change from $\frac{S}{mu}$ to $\frac{S}{ru}$, we need to multiply the former term by $\frac{mu}{ru}$, but this ratio depends on the box in which the species *S* resides. The value $\frac{S}{ru}$ is then rounded to the closest integer. If, as above, the deterministic model contains a transient signal, we discarded it. We took $\frac{s}{mu}\equiv s(t_{0})$, that is concentration to be converted is the value of species S at the beginning of oscillatory part of deterministic trajectory of S.

3 To convert the reaction rates from model units to real units we use the law of mass action and the conversion ratios found at step 2.

We will show how the method works using *L*_3_, the translation reaction to FRQ.

We have $f_{r}\overset{{\;L}_{3\;}}{\to }f_{r}+f_{p}$. Then $\frac{df_{p}}{dt}\;=\;L_{3}*f_{r}$.

Using model units we have

$$\frac{{df}_{p}/{mu}_{d}}{dt/hr}\;=\;\frac{hr}{{mu}_{d}}\;*\;L_{3}\;*\;\frac{f_{r}}{{mu}_{c}}\;*\;{mu}_{c}\;=\;L_{3}\;*\;hr\;*\;\frac{{mu}_{c}}{{mu}_{d}}\;*\;\frac{f_{r}}{{mu}_{c}}\;=\;L_{3,mu}\;*\;\frac{f_{r}}{{mu}_{c}},$$

where *hr* stands for hour, our unit of time, and *mu*_*d*_ and *mu*_*c*_ are the model unit of concentration for species in box *d* and *c*, respectively.

*L*_3,*mu*_ and $\frac{f_{r}}{{mu}_{c}}$ are the values of *L*_3_ and *f*_*r*_ expressed in model units. They are found from the deterministic model.

When *L*_3_ is expressed in molecular number units, we have

$$L_{3,ru}=\frac{L_{3}}{1/hr}=L_{3}*hr=L_{3,mu}*\frac{mu_{d}}{mu_{c}}=L_{3,mu}*\frac{{\left(mu/ru\right)}_{d}}{{\left(mu/ru\right)}_{c}}=3.02387\frac{890.1612}{5548.963}=0.4851.$$

where *L*_3,*ru*_ is the value of *L*_3_ expressed in molecular number units.

Likewise, the other reaction rates can be converted from model units to molecular number units using the deterministic values and the conversion ratios found in step 2.

Note that when converting the reaction rates, we keep the ratios $\frac{dS}{dt}$ the same. Here *S* is the concentration of a species. We do not change the qualitative behavior of the system by conversion, just express it in different measurement units.

### Method of determination of protein:DNA and RNA:DNA ratios specifying the scale of the stochastic model

The protein:DNA and RNA:DNA ratios in a cell were experimentally determined to set the scale parameters for the stochastic network. Protein, RNA, and DNA samples were extracted simultaneously from cultures of *Neurospora crassa*, strain FGSC 1858 “bd” (Fungal Genetics Stock Center, 4024 Throckman Plant Sciences Center, Kansas State University, Manhattan, KS 66506). The cultures were grown over 48 hours in the dark such that the total growth time was kept constant as previously described[45] under the “cycle 1” experiment. A kit from “Norgen Biotek Corporation,”(3430 Schmon Parkway, Throld, Ontario, Canada L2V 4Y6) was used to extract RNA, DNA and protein from the same sample. The kit used was Product # 47700, “RNA/DNA/Protein Purification Plus Kit.” Their protocol was followed, including the step 1F, for cell lysate preparation for fungi. Samples were done from thirteen different time points spaced at 4 hour intervals over 48 hours. A total of three preps were done for each time point, with a useable sample detected in 2–3 of the preps. The DNA and Protein amounts from each of these preps, were determined on a “Qubit 2.0 Fluorometer” instrument (ThermoFisher Scientific, 168 Third Avenue, Waltham MA 02451). The RNA concentration was determined using an Agilent BioAnalyzer RNA 6000 Nano chip (Agilent, Palo Alto, California). The amounts were converted to nanomoles [46], averaged, and then ratios RNA:DNA and DNA:Protein were calculated (Table 1).

### Stochastic simulation algorithm-direct method

For simulating exact trajectories of a network’s temporal evolution, we used a variant of Gillespie’s simulation algorithm called the direct method[13, 20]. Gillespie showed that knowing the state of the network at a time *t*, we can infer the exact distribution of the time of next reaction, *t* + *τ*, and the probability of each reaction taking place at time *t* + *τ*. Thus, we obtain an exact distribution of the state of the network at time *t* + *τ*. The Direct method uses these distributions to sequentially sample the time of the next reaction and the reaction that occurs next. It works as follows.

Given a set of parameters Θ = {*X*_1_(0), *X*_2_(0), …, *X*_*N*_(0), *k*_1_, …, *k*_*M*_}and a final time *T*, we do the following:

1. Initialize the system, i.e. set *t* = 0 and *X* = *x* = {*x*_1_(0), *x*_2_(0), …, *x*_*N*_(0)}.
2. Calculate the propensities, *a*_*j*_(*x*), *j* = 1, … *M*, and their sum $a_{0}\;=\;\sum_{j\;=\;1}^{M}a_{j}(x)$.
3. Draw the random time step value to the next reaction, *τ*, as an exponential random variable with mean 1/*a*_0_(*x*) and the next reaction. Draw the type of the next reaction to be executed, *j*_*next*_, as a discrete random variable with probabilities $\frac{a_{j}(x)}{a_{0}(x)}\;$, *j* = 1, … *M*,.
4. Update the state *X* assuming reaction $R_{j_{next}}$ took place. Update the time, *t* = *t* + *τ*.
5. If *t* < *T* go to step 2, else stop.

The Direct method yields a trajectory of the network state {*x*(*t*_0_), *x*(*t*_1_), … *x*(*t*_*k*_)} in the time interval [0, *T*]. The trajectory can be thought of as belonging to a single cell. We refer to this trajectory as a Gillespie trajectory (of a single cell). Here 0 = *t*_0_ < *t*_1_ < ⋯ < *t*_*k*_ < *T*with *t*_*i*_,i = 1,…,k, being the reaction times of the reactions that fire before *T*. Such a trajectory completely identifies the network state at any time in the interval [0, *T*].

### The fitting method

To analyze the initial behavior of the clock network we collected data on a *CCG* protein from 868 single cells. The fluorescence level of the *CCG* protein in each cell was recorded every half hour for 10 days[13].

As a first pass, we constructed a normalized periodogram for each cell, and then we calculated the average (over 868 cells) of these 868 periodograms. Through normalization we made the sum of normalized periodogram values equal to 1. Normalization enabled us to use periodogram values that are invariant to scaling[13]. We did not use this periodogram normalization in the more sophisticated analysis of the 1591 single cell data set where we applied a bias-correction to the average observed periodogram (See section below on removing the detection noise).

At the level of millions of cells the level of *CCG* is circadian, i.e. cyclical with a period of ~24 hours. To obtain stationary time series we used the moving average method to remove the 24-hour linear trend from the original time series. The periodograms were calculated on detrended data[13].

We assume the average periodogram describes the dynamical behavior of *CCG* protein because it captures the periods and amplitudes in the system.

We selected this summary statistic in fitting the stochastic network because we looked for models with periodic behavior at the single cell level. This choice was first proposed to describe stochastic oscillatory networks near their Hopf bifurcation (*i*.*e*., a point in the parameter space where oscillations first appear), and the use of the periodogram as the statistic driving the fitting was successfully used in this context[36]. The periodogram captures two important features of an oscillation, amplitude and period. As we expect the oscillatory trajectory to be a mixture of sinusoids with only few of them being relevant, we think the important features of an oscillatory trajectory are embedded in its periodogram. Also, unlike other methods that try to match individual trajectories produced by a stochastic model[23] [47], we try to fit the average of these periodograms. Our view is that to compare two stochastic models it is better to use summary statistics that relates to an average of the stochastic trajectories rather than comparing individual trajectories for four reasons. One, the individual stochastic trajectories are very noisy. Two, averaging the periodogram of individual trajectories reduces this noise. Three, this fitting approach has already proven successful[36]. Four, fitting using 1000s of individual trajectories by the method of maximum likelihood has not proved computationally tractable.

### Markov Chain Monte Carlo (MCMC) methods

We used MCMC methods [13, 27, 28] to find sets of parameters in the stochastic network (Fig 1) that best describe the observed average periodogram of a collection of cells. In each Monte Carlo update we used Gillespie’s direct method to simulate 1024 Gillespie trajectories of the system state using a given set of parameters. Here the parameters are the 12 initial molecular numbers and 22 reaction rates as described earlier in Materials and Methods. We calculated the average periodogram of simulated trajectories and determined how well it matched the cell average periodogram. Then we updated a randomly chosen parameter using the Metropolis–Hastings algorithm. The 1024 simulated trajectories were run in parallel on a GPU.

The ensemble ℚ used to fit the average periodogram is

$$Q(\Theta )\;=\;\Omega^{-1}\prod_{f}\frac{1}{\sqrt{2\pi \sigma_{f}^{2}}}exp\left(-\frac{{\left(\bar{Q_{f}^{cell}}-\bar{Q_{f}^{sim}}\right)}^{2}}{2\sigma_{f}^{2}}\right)\;=\;exp(-\chi^{2}/2)\Omega^{-1}\prod_{f}\frac{1}{\sqrt{2\pi \sigma_{f}^{2}}}$$ (1)

where $\;\bar{Q_{f}^{cell}}\;and\;\bar{Q_{f}^{sim}}$ are average periodogram values of cell and simulated trajectories, respectively, calculated at frequency *f*. The parameter $\sigma_{f}^{2}$ is the variance of cell periodogram values at frequency *f* and is determined experimentally by bootstrapping the periodograms of single cells. The form of the likelihood entails invoking the Central Limit Theorem. The associated *χ*^2^ = −2*ln*ℚ + *constant*.

We initialized our Markov chains with working oscillatory networks describing the clock at the macroscopic level of 10^7^ cells determined previously by the ensemble method[28]. For each chain, we took a set of parameters from the deterministic ensemble family given in Yu et al.[28] and converted it to a set of stochastic parameters as described above.

**Fig 2:**
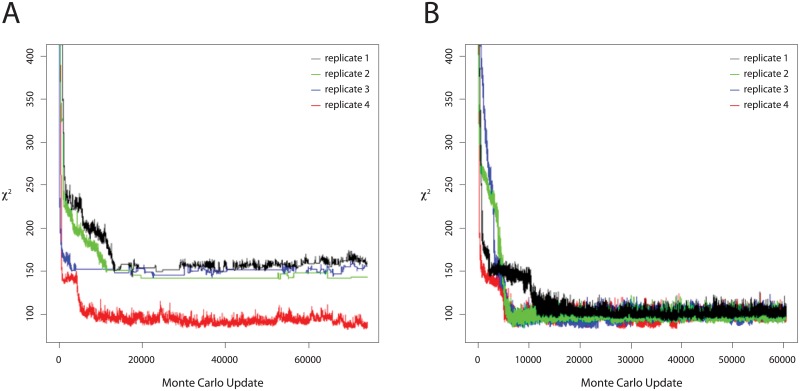
Monte Carlo simulations used for fitting average periodogram of the 868 single cell data. (A) 4 chains run using Metropolis-Hastings algorithm. (B) 4 chains starting with the same parameters, but run using Parallel Tempering algorithm. No bias-correction was applied (see Materials and methods).

To make sure we are covering a broad region of the parameter space, we started 4 Monte Carlo chains, each one with a different set of parameters. After running these Monte Carlo chains for about 74,000 iterations we ended up with chi-squared values of different levels, see Fig 2A.

We concluded that each of these chains might have been trapped to a different local minimum. To avoid being stuck at a local minimum we introduced an alternate MCMC method, the parallel tempering algorithm[48, 49]. Each set of parameters used to start a Metropolis-Hastings chain was now used to start a parallel tempering algorithm. **(see** Materials and methods**).**

Our Metropolis-Hastings algorithms were designed as random walk algorithms.

The target density was ℚ. At iteration *k* we randomly picked a parameter $x_{i}^{k}$ of the parameter set $x^{k}\;=\;(x_{1}^{k},x_{2}^{k},\ldots ,x_{34}^{k})$ and updated it using a uniform proposal kernel $U(x_{i}^{k}-\alpha_{i},x_{i}^{k}+\alpha_{i})$, i.e. the proposal density was $q(x_{i}^{k+1}|x_{i}^{k})\;=\;\frac{1}{2\alpha_{i}}I_{\left(x_{i}^{k}-\alpha_{i},x_{i}^{k}+\alpha_{i}\right)}(x_{i}^{k+1})$.

The proposed parameter set was $y^{k+1}\;=\;(x_{1}^{k},x_{2}^{k},\ldots x_{i}^{k+1},\ldots ,x_{34}^{k})$. Then, we set

xk+1={yk+1withprobabilityρ(xk,yk+1),xkwithprobability1-ρ(xk,yk+1)},

where *ρ*(*x*, *y*) = *min*{1, *L*(*y*)/*L*(*x*)}.

It is well known that for random walk Metropolis-Hastings algorithms the step-widths *α*_*i*_ must be fine-tuned to ensure the chain is converging in a manageable time. While we tried to optimize the choice of the *α*_*i*_′*s*, we noticed that it might take too long for some chains to converge (Fig 2A). Parallel tempering algorithm avoids the calibration of these 34 hyperparameters.

### Parallel tempering as an ensemble method

The idea of a parallel tempering algorithm is to simulate *K* replicas of the original system, each replica being simulated at a different temperature. So, each replica is a Markov chain having a tempered target distribution of the form

$$Q_{T}(\Theta ){\;=\;\Omega }^{-1}\prod_{f}\frac{1}{\sqrt{2\pi {T\sigma }_{f}^{2}}}exp(-\frac{{\left(\bar{Q_{f}^{cell}}-\bar{Q_{f}^{sim}}\right)}^{2}}{2{T\sigma }_{f}^{2}})\;=\;exp(-\chi^{2}/2)\Omega^{-1}\prod_{f}\frac{1}{\sqrt{2\pi \sigma_{f}^{2}}}$$

For high “temperature” *T*, the peaks of ℚ_*T*_ become flatter and broader, making the distribution easier to sample via MCMC methods. High-temperature replicas can sample large volumes of parameter space, whereas low-temperature chains are usually sampling from a local region of the parameter space which may trap them to a local minimum. Parallel tempering achieves superior results by allowing different replicas to exchange their states. Thus, high-temperature replicas ensure that lower temperature chains can access different regions of the parameter space.

The way that a parallel tempering run is set up is as follows. To a set of K replicas we assign temperatures from a grid *T*_1_ < *T*_2_ < ⋯ < *T*_*k*_, with *T*_1_ = 1 corresponding to our target replica. Each replica explores its tempered distribution using an MCMC method. After a predetermined number of in-chain iterations, swaps between usually adjacent replicas are proposed. A proposed swap between replicas at temperatures *T*_*i*_ and *T*_*j*_ is accepted with probability

$$\rho_{ij}=min\left\{1,\frac{{\mathbb{Q}}_{T_{i}}(x_{\left(j\right)}){\mathbb{Q}}_{T_{j}\left(x_{\left(i\right)}\right)}}{{\mathbb{Q}}_{T_{i}}(x_{\left(i\right)}){\mathbb{Q}}_{T_{j}\left(x_{\left(j\right)}\right)}}\right\}$$

where *x*_(*i*)_ is the state of *i*^th^ replica. When a swap is accepted, the replicas exchange their positions in the parameter space; replica *i* takes configuration *x*_(*j*)_ *a*nd *j* assumes the position at *x*_(*i*)_. Since hottest replicas can sample big regions of the parameter space, then, if their locations propagate to the coldest replica, they can help it explore different regions of parameter space. Thus, the goal in choosing an effective grid of temperatures is to make sure the hottest replicas can freely explore the parameter space, i.e choose *T*_*k*_ big enough, and to choose the intermediate temperatures in such a way that *ρ*_*i*,*j*_′*s* are big enough to allow each replica to easily move between configurations sampled at different temperatures.

### Choosing the grid (K) and temperatures in parallel tempering

Now we show how we chose K, the number of replicas, T_K_, the maximum temperature and the temperature grid *T*_1_ < *T*_2_ < ⋯ < *T*_*k*_.

Our method is based on the procedure described previously[50].

First, we chose the number of replicas $K\;=\;\sqrt{d}$, where d is the number of components of *θ*.

Then we chose maximum temperature *T*_K_.

We took $T_{k}\;=\;\frac{\chi^{2}(\theta^{0})}{30}$, i.e. we divided the chi-square value of our initial parameter set *θ*^(0)^ by 30. The hottest replica will start with a chi-square value of 30. We wanted the hottest replica to have a high in-chain acceptance rate while having a not too flat distribution. The number of data points used in calculating chi-square values was 85.

Then we ran Metropolis-Hastings for a replica with this temperature for 200 iterations. If acceptance rate was outside the range (0.6, 0.75), then we changed *T*_*K*_ and ran M-H again for 200 iterations. We did this until the acceptance rate fell within the range (0.6, 0.75).

We made a linear grid with K temperatures and set a target swap rate of 0.4 for any two neighboring replicas. Then we run the parallel tempering in the following way:

1. every replica does an update of its parameter set *θ*.
2. attempt swaps between replicas 1 and 2, 3 and 4, 5 and 6,…
3. attempt swaps between replicas 2 and 3, 4 and 5, 6 and 7,…
4. repeat steps 1), 2) and 3) 200 times

For every pair of neighboring replicas (*i*, *i+1*) we calculated

$$Q_{i,i+1}\;=\;\frac{1}{N_{swap}^{i,i+1}}\sum_{l\;=\;1}^{N_{swap}^{\left(i,i+1\right)}}ln(\rho_{i,i+1}^{l}),$$

where $N_{swap}^{\left(i,i+1\right)}$ is the number of attempted swaps between replicas *i* and *i+1* and $\rho_{i,i+1}^{l}$ is acceptance probability of the 𝑙^𝑡*h*^ attempt at swapping *i* and *i+1*.

If $R_{i,i+1}\;=\;\left[\sqrt{\frac{Q_{i,i+1}}{ln(0.4)}}\right]>0$, then we add to the grid *R*_*i*,*i*+1_ temperatures, evenly spaced between *T*_*i*_ and *T*_*i*+1._

We run this add-temperature process 3 times to make sure we have enough temperatures in the grid.

Then we shifted the temperatures between *T*_1_ and *T*_*k*_ as follows.

1. Run parallel tempering with the new temperature set doing the above steps 1), 2) and 3) 350 times.
2. For each temperature *T*_*i*_ calculate the flow fraction
  $$f(T_{i})\;=\;\frac{n_{up}(T_{i})}{n_{up}(T_{i})+n_{down}(T_{i})},$$
  where *n*_*up*_(*T*_*i*_) and *n*_*down*_(*T*_*i*_) is the total number of replicas that were drifting upward, respectively downward, when they visited *T*_*i*_.
3. Linearly interpolate *f* between temperatures
4. Calculate *g* the inverse function of *f*
5. Change the temperature values from *T*_*i*_ to $T_{i}^{new}\;=\;(1-\;\frac{i-1}{K-1})$.

This process of shifting the intermediate temperatures was repeated 3 times.

The shifting of temperatures was done to optimize the flow of replicas through the temperature grid.

After that, we ran the parallel tempering algorithm for about 60,000 Monte Carlo updates, where by update we mean the steps 1), 2) and 3) described in the add-temperature process. Some of the control parameters and summary statistics for the MCMC runs are also summarized in S1 Table.

### Removing the detection noise from the average periodogram

A model to calculate the contribution of detector noise to the periodogram variance was derived under mild assumptions, and the detector noise, propagated to the periodogram in the supplement[13]. The assumptions in this calculation were that the total noise could be decomposed additively into stochastic intracellular noise and detector noise, that the stochastic intracellular noise component is independent of the detector noise component, and that the detector noise in the Rhodamine B level measurements used in normalization of fluorescence measurements can be neglected[13]. The detector noise was independently quantified by replacing cells with fluorescent beads in the microfluidics experiments. In this way a universal system of measurement of gene expression at the single cell level was developed and used here[13].

Suppose we observe the fluorescence values in an experiment with *K* cells and *L* equidistant observation times $t_{j}\;=\;(j-1)\frac{T}{L}$. Here *j* = 1, …, *L* and *T* is the duration of the experiment. Denote the frequencies of interest by $f_{l}\;=\;\frac{l}{T}$, *l* = 0,…, [*L*/2]. Also assume the cells are treated with rhodamine B to reduce experimental noise.

Then, the detector noise contribution to the periodogram variance at frequency *f*_*l*_ is given by[13]:

$${\left(\sigma_{l}^{e}\right)}^{2}\;=\;\frac{2\sigma_{\epsilon }^{2}}{KL}[\langle Q(f_{l})\rangle \gamma_{Q}\left(l\right)+Re(\langle R(f_{l})\rangle {\beta_{Q}\left(l\right)}^{*})]-\frac{\sigma_{\epsilon }^{4}}{KL^{2}}\left[{\left|\gamma_{Q}(l)\right|}^{2}+{\left|\beta_{Q}(l)\right|}^{2}\right],$$

where 〈*Q*(*f*_*l*_)〉 and 〈*R*(*f*_*l*_)〉 are the population means of, respectively, average periodogram and average squared Fourier transform of the observed rhodamine B-normalized, detrended fluorescence time series. The quantity $\sigma_{\epsilon }^{2}$ is the variance of the fluorescence signal due to the detector noise averaged over all cells and time points. This variance was determined experimentally by varying the incident light intensity and measuring the resultant variance in fluorescence of fluorescent beads replacing cells in a microfluidics experiment identical to that used for cells[13]. The quantities *γ*_*Q*_(*l*) and *β*_*Q*_(*l*) are functions of the weights used in the moving-average detrending process[51], a standard for the literature. They do not depend of the observed fluorescence signals.

To compare the simulated average periodogram values with observed average periodogram values we need to remove the bias due to detection error.

The bias formula is given by

$$Q^{bias}(f_{l})\;=\;\frac{\sigma_{\epsilon }^{2}}{L}\gamma_{Q}(l).$$

When we try to fit the cell average periodogram, we compare *Q*(*f*_*l*_) − *Q*^*bias*^(*f*_*l*_) to *Q*^*model*^(*f*_*l*_), with *Q*(*f*_*l*_) being the average periodogram over *K* cells calculated at frequency *f*_*l*_ and *Q*^*model*^(*f*_*l*_) being the average periodogram of the simulated time series calculated at *f*_*l*_. Unlike the analysis of the 868 single cell data set, the analysis of 1591 single cell data set with the bias correction does not normalize the periodograms. The whole fitting process is done on an absolute scale without periodogram normalization.

The ensemble to fit the average periodogram becomes

$$Q_{bias-free}(\Theta )\;=\;\Omega^{-1}\prod_{l}\frac{1}{\sqrt{2\pi \sigma_{f_{l}}^{2}}}exp(-\frac{{\left(Q(f_{l})-Q^{bias}(f_{l})-Q^{model}(f_{l}\right)}^{2}}{2\sigma_{f_{l}}^{2}})\;=\;exp(-\chi^{2}/2)\Omega^{-1}\prod_{l}\frac{1}{\sqrt{2\pi \sigma_{f_{l}}^{2}}}$$ (2)

with ${\left(\sigma_{f_{l}}^{c}\right)}^{2}\;=\;\sigma_{f_{l}}^{2}-{\left(\sigma_{f_{l}}^{e}\right)}^{2}$. The Central Limit Theorem is being invoked to obtain the approximate distribution of the average periodogram over > 1000 single cells needed to write down the ensemble in (2). See [13] for details.

## Results

### Parallel tempering as opposed to Metropolis Hastings (M-H) Monte Carlo is sufficient for fitting stochastic models with many parameters

As seen in Fig 2B the parallel tempering algorithms greatly improved the mixing of the chains and helped them escape local minima. These algorithms also converged much faster when compared to M-H algorithms. Their only downside is that they are slightly more computationally intensive and in general take longer to run when compared to the simple Metropolis-Hastings algorithms. However, parallelization can greatly reduce the additional time taken to run a parallel tempering algorithm when compared to Metropolis-Hastings algorithm. Better mixing and faster convergence more than compensate for the longer run time of each iteration.

For the chains in Fig 2A the average computation time per iteration were 25.76, 3.62, 0.33 and 2.41 seconds, respectively. For the four chains in Fig 2B the computation times per iteration were on average 41.98, 34.07, 30.11 and 16.89 seconds, respectively.

We see that, when using Metropolis-Hastings algorithm, computation time, convergence and mixing varies greatly with the initial parameters in Fig 2A.

Even though the computation time when using parallel tempering algorithm was on average longer compared to the time used by the Metropolis-Hastings algorithm, we see that the parallel-tempering chains quickly set to what is likely the region of the parameter space with lowest chi-squared value. Beginning with iteration 20,000 the chi-square value varied between 82.87 and 125.5 for all chains. The mixing of these chains was excellent, with the swap acceptance rate between replicas at neighboring temperatures being larger than 0.5 for all parallel-tempering chains. The maximum number of replicas used in a parallel-tempering algorithm was 7. The maximum temperature was 10.

**Fig 3:**
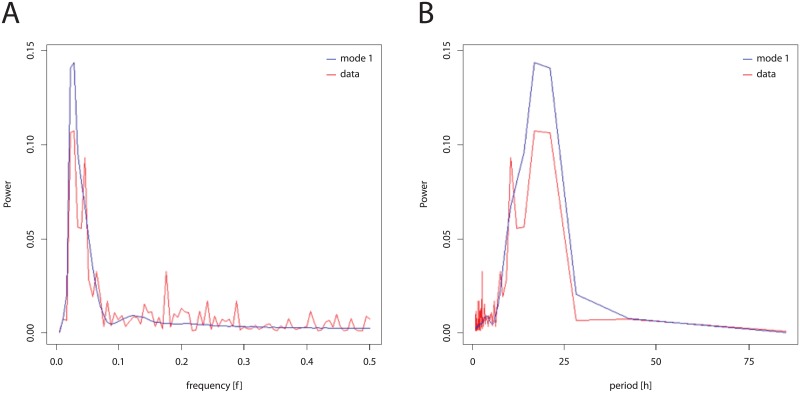
Fitting of average periodogram of the cells (red) by average periodogram produced by best parallel-tempering model(blue). (A) in frequency domain (B) using period.

The best model given by the parallel-tempering algorithm gave a good fit to the average periodogram of the cells as can be seen in Fig 3. The model captures the main frequencies in the data. It does not fit the data very well at high frequencies, but at high frequencies data are very noisy.

### The fit of the stochastic model can be improved substantially by increasing the number of cells and subtracting the detection noise from the periodogram

To reduce the noise in the periodogram in Fig 3 we increased the number of isolated cells to 1591 in a replicate experiment and removed the detection noise in the periodogram. See Table 2. Without normalization of the periodogram the final *χ*^2^ was 671.332 as opposed to 12,024.9, when the bias correction was not made. With 240 data points and 34 parameters, the chi-squared contribution per data point was 2.80, which is comparable to earlier work on a macroscopic scale[45].

**Table 2:** Ensemble means and standard errors indicate that the parameters in stochastic network for single cells are tightly specified by the new fitting method. The parameters including the initial numbers of molecules and the rate constants in Fig 1 are labeled in the first column. In the second column are the initial parameter values used from a deterministic model ensemble[28], in which WC-2 constant over time was used to initialize both the Metropolis Hastings MCMC runs (Fig 2A) and the parallel tempering MCMC runs (Fig 2B). In the third column the parameters from the deterministic model ensemble are converted into units appropriate for the stochastic network as described in Materials and Methods. The last four columns are the ensemble means and standard errors (across the ensemble) generated by parallel tempering (see Materials and methods) for a single cell experiment with 868 single cells or 1591 single cells.

Parameter	Initial_Parameter value from MCMC Deterministic model ensemble (Yu et al., 2007)	Initial Parameter values from Deterministic model ensemble (column 2) re-scaled to_molecular number_units of stochastic network (column 3)	Mean parameter values from model ensemble computed by Parallel tempering_	Standard error (SE) of parameter value across ensemble computed by parallel tempering	Mean parameter values from model ensemble computed by Parallel tempering_	Standard error (SE) of parameter value across ensemble computed by parallel tempering
Number of cells	-	-	868	868	1591	1591
u_r0	3.99924	113	5015.225951	39.46834951	2156.705728	68.14603254
u_r1	0.442441	18	5427.14616	38.81394987	22.46137677	0.872953544
u_p	4.24E-07	459	5052.398397	39.86454551	2144.149238	68.74768856
f_0	0.356365	1	0.498322148	0.006827531	0.465055176	0.01143672
f_1	0.0824576	0	0.501677852	0.006827531	0.534944824	0.01143672
f_r	4.90E-06	31	5099.803691	39.65320564	59.15869679	2.637452857
f_p	3.0804	345	5661.033184	37.79536413	2534.336311	77.72114325
w	9.24126	101	5070.154735	39.25742344	55.40042039	1.674320488
g_0	0.0066195	1	0.498508576	0.006827539	0.71623752	0.010337149
g_1	2.59E-06	0	0.501491424	0.006827539	0.28376248	0.010337149
g_r	1.17E-06	26	3086.66182	44.02007694	35.67840252	1.030258983
g_p	1.37E-05	102	5272.938106	41.68301079	59.19075145	4.920903774
A	0.000658482	6.06E-13	1.84E-10	3.59E-12	2.56E-10	7.31E-12
Abar	0.546986	0.546986	50.03130674	0.39493562	1.589532708	0.035661845
S1	0.061594783	83.70771546	75.50835675	0.195849227	80.12566921	0.302471515
S3	0.00146575	3.569116497	10.79752752	0.13705406	0.400641074	0.036565894
S4	2.2396	5453.449297	8229.792075	51.85745823	8316.020583	100.2852188
D1	0.723678	0.723678	11.6411168	0.125746972	1.294999006	0.030289616
D3	0.299703	0.299703	13.54885771	0.126263685	4.382612039	0.181101578
C1	0.0428595	4.81E-05	0.002859534	2.05E-05	0.000932789	2.47E-05
L1	31.7758	4.244678204	11.73643559	0.073999739	4.777735371	0.106626479
L3	3.02387	0.485087349	1.339312652	0.010385343	0.665600817	0.011127036
D4	0.00323262	0.00323262	0.077027847	0.001450665	0.08474029	0.004700587
D6	0.15183	0.15183	4.114778877	0.068424489	0.193685712	0.002236097
D7	0.138387	0.138387	3.008652124	0.046190832	2.130911791	0.090030385
D8	0.00248668	0.00248668	0.159664546	0.00266928	0.007744621	0.000182717
C2	0.162687246	0.162687246	6.323785664	0.069435513	1.515554675	0.077548547
P	19.5648	3.12E-11	1.15E-09	1.88E-11	2.72E-09	4.83E-11
Ac	4.06813	7.82E-09	2.68E-07	4.65E-09	1.86E-08	2.55E-09
Bc	2.52197	2.52197	66.30689649	0.666502458	2.581096866	0.040197442
Sc	1.01E-06	73.80414613	1097.22398	6.307615581	61.51499414	1.109629713
Lc	1.15E-08	2.231095711	3.743285409	0.038176388	1.61524392	0.017335914
Dcr	0.219758	0.219758	0.625739758	0.02113632	0.150810052	0.00291715
Dcp	0.696903	0.696903	1.982857699	0.007835124	0.54063952	0.006141903

### There are strong similarities in the rate constants between the stochastic clock network and deterministic clock network

The ensemble averages of parameters and their standard errors across the ensemble are reported in Table 2 and in bar charts in supplementary S1 Fig. Some of the parameters are key to sustained oscillations in the deterministic model[28]. Some of these include the activation (A) and deactivation rate(Abar), the decay rate of the stabilized *wc-1* mRNA (D7)[28], the decay rate (D6) of the FRQ protein[52, 53].

The initial parameter values were computed by MCMC Metropolis Hastings Method[28] for a deterministic model, in which the protein WC-2 was treated as constant to good approximation. The best parameter values in this ensemble were then converted to the molecular number units of the stochastic network in Table 2 (column 3). For example, in the stochastic network the initial numbers of molecules in each cell in Table 1 are given as opposed to concentrations used in the deterministic model. This conversion is described in Materials and Methods. Generally there is good agreement between the estimated rate constants estimated from the trajectories of 1,591 cells (column 6) and the initial guess from the deterministic model (column 3), but no such agreement exists for the smaller experiment with only 868 single cell trajectories. All discussion below is for the larger single cell experiment with 1,591 cells. In this discussion below *wc-1* and *wc-2* and their products are positive elements in the clock, while *frq* and its products are negative elements providing negative feedback to *wc-1* and *wc-2* and their products[35] in Fig 1.

The decay rate of FRQ, D6, is thought to determine the period of the clock oscillator[52] and be involved in the phenomenon of temperature compensation in the clock. As the FRQ decay rate D6 decreases, the period is expected to increase. This coupling of period and FRQ may be more complicated[53]. The stochastic network’s decay rate (.194 +/- .002) is in quite good agreement with the macroscopic deterministic model (0.152).

The decay rate of the stabilized *wc-1* mRNA, D7, in Fig 1 is thought to be critical determinant of clock oscillations[28]. The theory predicted (and experiment confirmed in previous work[28] that there should be small decay rate or a long half-life at the macroscopic level. The decay rate D7 in the stochastic network (2.131 +/- 0.090) appears somewhat higher than measured in the deterministic model (0.138). One possible explanation is that the constraint on decay rates for isolated cells that experience stochastic intracellular noise in phase may be relaxed relative to that in a deterministic model at the macroscopic level. If the oscillations are actually supported by the noise, by way of some Stochastic Resonance mechanism[38], then this may impose less severe constraints on the decay rate D7.

Another critical parameter for oscillations to occur in the deterministic model is the activation (A) and deactivation rates (Abar) of the oscillator *frq* gene by WCC in Fig 1. These activation and deactivation rates in the stochastic model (2.56E-10 +/- 7.31E-12 and 1.590 +/- 0.036) tend to be qualitatively similar (6.06E-13 and .0.547), both being quite small.

Another critical parameter for oscillations in the deterministic model is the rate of deactivation of WCC (the P reaction) by FRQ [28]. The deactivation rate (P) in the stochastic model (2.7E-9 +/- 4.8E-11) is similar to that estimated at the macroscopic scale (3.12E-11), both being small. To assist in comparing the fitted macroscopic model with the fitted single cell stochastic model, bar charts of the parameters in Table 2 can be found in supplementary S1 Fig.

The new MCMC method for specifying the parameters from periodogram tends to produce standard errors across the ensemble that are one to two orders of magnitude smaller than the ensemble means. The parameter values are quite tightly specified by the new estimation method and the use of at least 1,500 cells.

**Fig 4:**
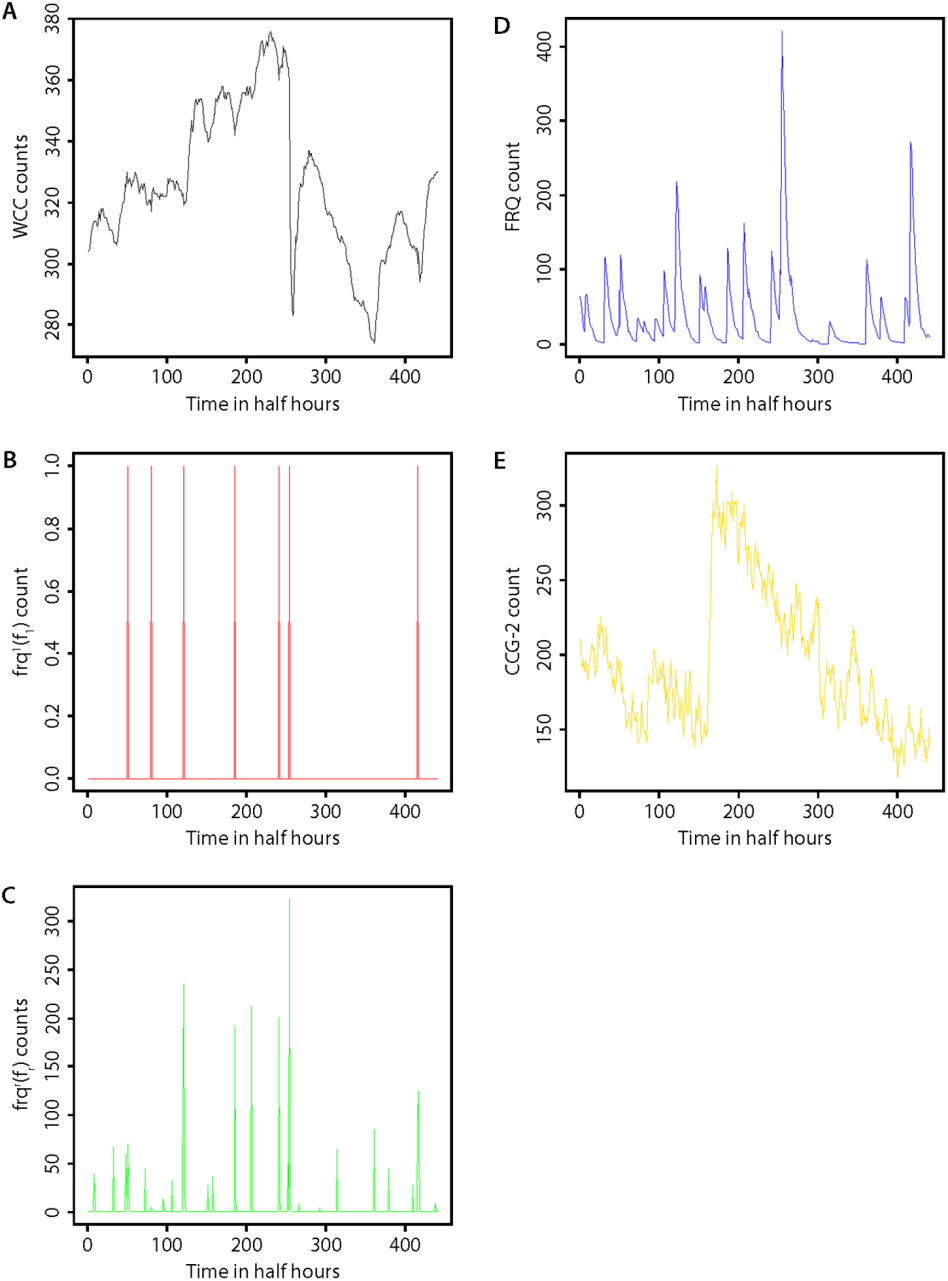
Multiple views of one stochastic trajectory for one of the best fitting models from the 1591 cell data set. The Gillespie trajectory shown in part is derived from a best fitting model in Table 2 after bias-correction. The chi-squared statistic for this fitted model was 671.332. Time 0 actually corresponds to 20 h, and the last time point, to 240 h. (A) Trajectory of the WCC count. (B) Trajectory of the active *frq* gene count f_1_; the gene is either on or off; (C) Trajectory of the *frq* mRNA count f_r._ (D) Trajectory of the FRQ protein count. (E) Trajectory of the CCG-2 protein count.

It is interesting to see what this model actually looks like. Various views of a single Gillespie Trajectory are shown for one of the best fitting models (Fig 4). In panel A is shown the molecular counts of the positive element WCC. The counts are correlated with the activation of the FRQ gene in panel B, but the correlation is not perfect. Switching on the *frq* gene is a stochastic event in this model. The result of switching on the *frq* gene is transcriptional bursts in its mRNA in panel C. There are at least 9 such bursts in Panel C over a 240 h interval. The width of these bursts as shown is wider than the time that a *frq* gene is active in a cell. In turn there are resulting even broader peaks in the FRQ protein production in panel D. The noisiest trajectory is what we actually see, CCG-2 in panel E. These views of a Gillespie Trajectory give us a picture of the noise in a single cell under one fitted model in the model ensemble.

### The stochastic intracellular noise level (i.e., the size of the cell) can be experimentally determined as a parameter in the model

**Fig 5:**
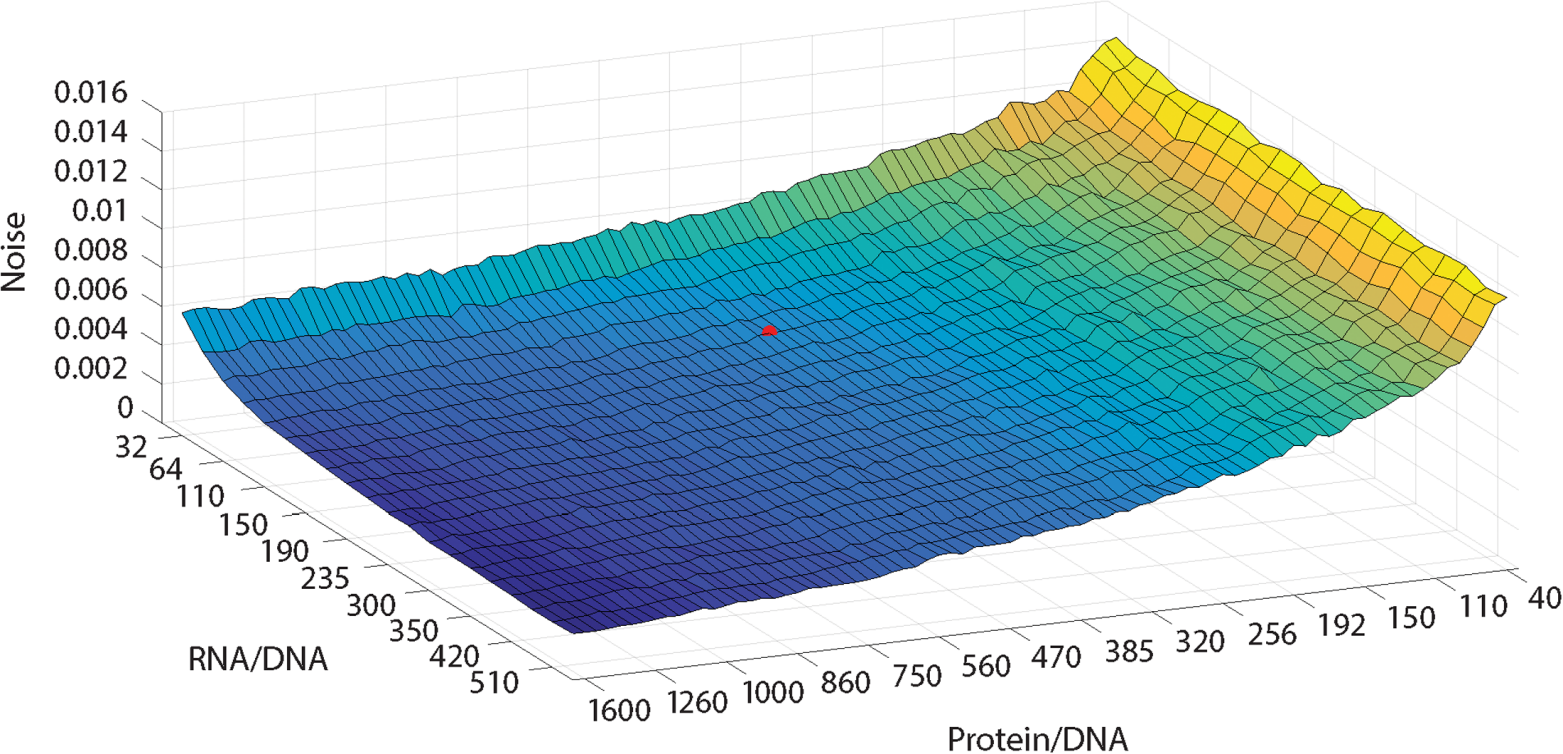
Stochastic noise in CCG-2 as a function varies systematically with hypothesized ratios of RNA/DNA and protein/DNA ratios within a single cell. The total stochastic noise $\sigma_{f}^{2}$ averaged over frequencies (f) in CCG-2 expression is computed from bootstrapping the 1024 Gillespie trajectories. The red dot denotes the experimentally determined ratios (see Table 1) and corresponds to a RNA/DNA and protein/DNA ratio of 128.7 and 412, respectively. The model ensemble used is described in Table 2; the model selected was one with minimum chi-squared statistic based on the Likelihood in Eq (1) for 868 single cells.

The mRNA to DNA ratios and protein to DNA ratios in conidia have been previously determined to be ~1:18:50[54]. Our ratios from Table 1 tend to be higher as 1:129:412, although the protein/RNA ratio is similar to previous reports. Here we report a higher amplification in the DNA -> RNA step of the Central Dogma. These ratios were then used to set the noise in the stochastic network (Fig 5). The network was subdivided into independent blocks that were only linked by catalytic reactions. Then the ratios in Table 1 were used to convert concentrations in the deterministic model into molecular numbers within the cell (as described in Materials and Methods for each independent block) as illustrated in Table 2.

The ratios of RNA to DNA and protein to DNA set the level of noise in the Gillespie trajectory of CCG-2. As the ratios get smaller the noise increases in the expression of CCG-2 in the Gillespie model trajectories for CCG-2 protein. The level of stochastic intracellular noise is then set by the amplification at each step in the Central Dogma.

**Fig 6:**
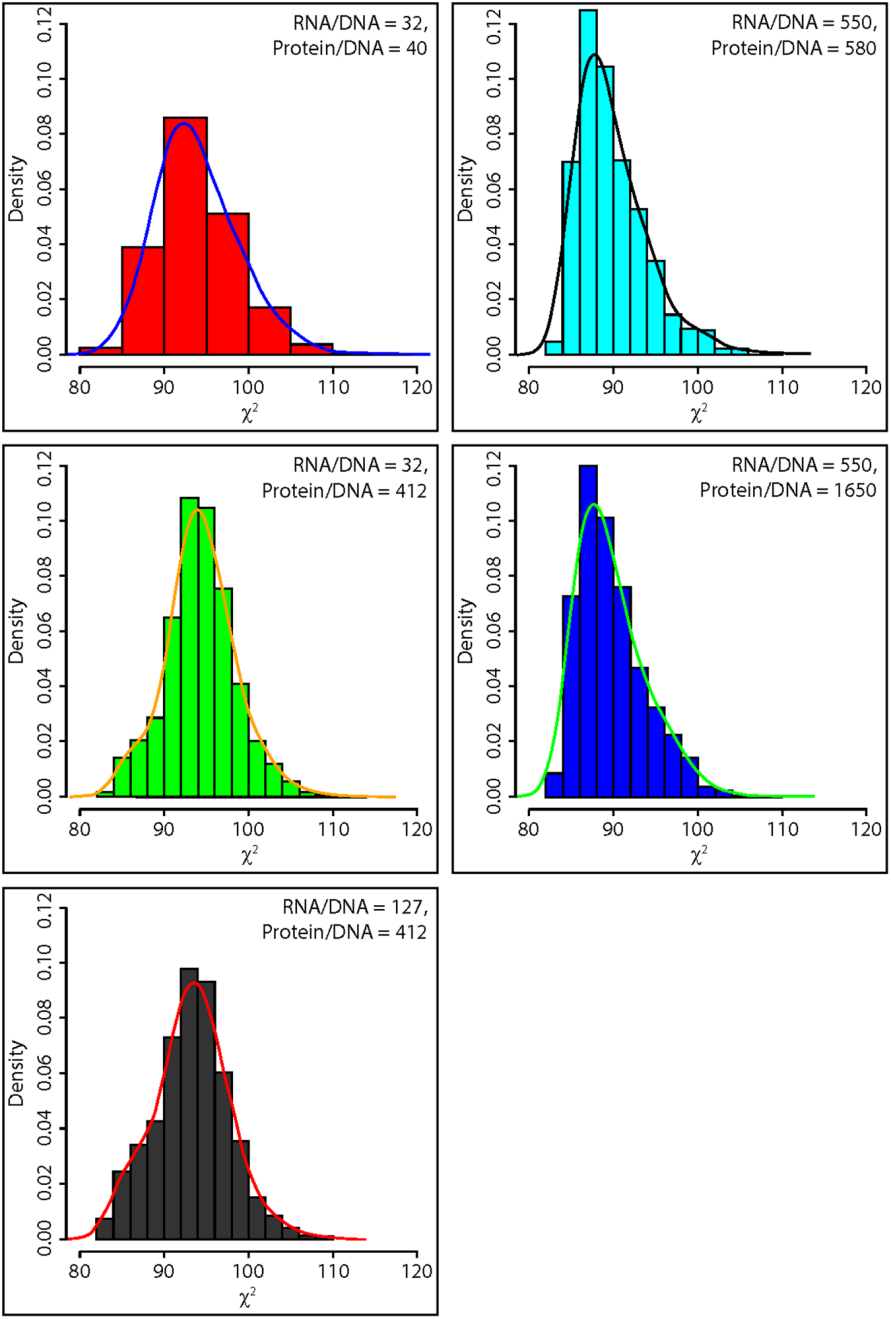
The fit of ensemble of models (*χ*^2^) is robust to variation in the RNA/DNA and protein/DNA ratios. Each ensemble had at least at least 1,400 models derived from an accumulation run. The equilibration runs were done with parallel tempering as described in the Materials and Methods. A smooth interpolation is provide for each histogram. The ensembles were derived from L in Eq (1) for 868 single cells with no bias correction.

Having experimentally determined these ratios, it is natural to ask how these ratios affect the goodness of fit of the model (Fig 6). We varied the ratios about the experimental values. A slightly better fit could be obtained by allowing the protein/DNA ratio to be slightly higher. The values of the chi-squared statistics across an ensemble would suggest that the fit of the model ensemble if fairly robust to variation in these ratios.

The robustness of the ensemble across different RNA/DNA and protein/DNA ratios can be understood by the fact that our stochastic models can be well approximated by a chemical Langevin equation, which in turn can be approximated by the ODE equations of the deterministic model. The chemical Langevin equation(CLE)[10, 55] is a stochastic equation that describes the rate of change of the state vector of molecular numbers, *X*, as follows:

$$\frac{dX(t)}{dt}\;=\;\sum_{j\;=\;1}^{M}v_{j}a_{j}(X(t))+\sum_{j\;=\;1}^{M}v_{j}\sqrt{a_{j}(X(t))}\Gamma_{j}(t),$$ (3)

The molecular numbers comprised in *X* are treated as continuous random variables. The first term on the right-hand side is just the rate function of the corresponding deterministic model, and the second term represents the noise due to the stochasticity of the reaction events. The *v*_*j*_ is the vector of changes in molecular numbers produced by the firing of reaction *R*_*j*_ and Γ_*j*_′s are statistically independent Gaussian white-noise processes. The crucial point here is that the strength of the noise term in the CLE *increases relative to* the deterministic rate term in the CLE, as the molecule numbers for RNA and protein *decrease*.

The change of RNA/DNA and protein/DNA ratios in Fig 5 was effected by rescaling the RNA and protein numbers in such a way that the corresponding deterministic model remained unchanged and only the noise term in the CLE was affected. So, if the deterministic term dominates the stochastic term, the noise level will not matter, hence the robustness.

### The Hilbert phase variation between cells provides an independent test of the goodness of fit of the model to the average of the observed periodograms of single cells

There are three quantities that characterize the periodic behavior of single cells, their period, amplitude, and phase[13]. Two of these quantities, period and amplitude, are captured in the periodogram used for fitting the model ensemble (Fig 3). The phase is functionally independent of the periodogram and hence independent of the first two quantities[13]; therefore, the phase can be used as a test of the adequacy of the model. The phase is not used in the fitting (Fig 3). The Hilbert phase can be calculated for each single cell trajectory and each Gillespie trajectory and measures the amount of cycles completed in a fixed period of time (and hence is a function of time). So, for example, 4 tires on a car would complete the same number of cycles in a fixed period of time and be in phase. This phase measure does vary with time and is a well known measure of phase[56].

**Fig 7:**
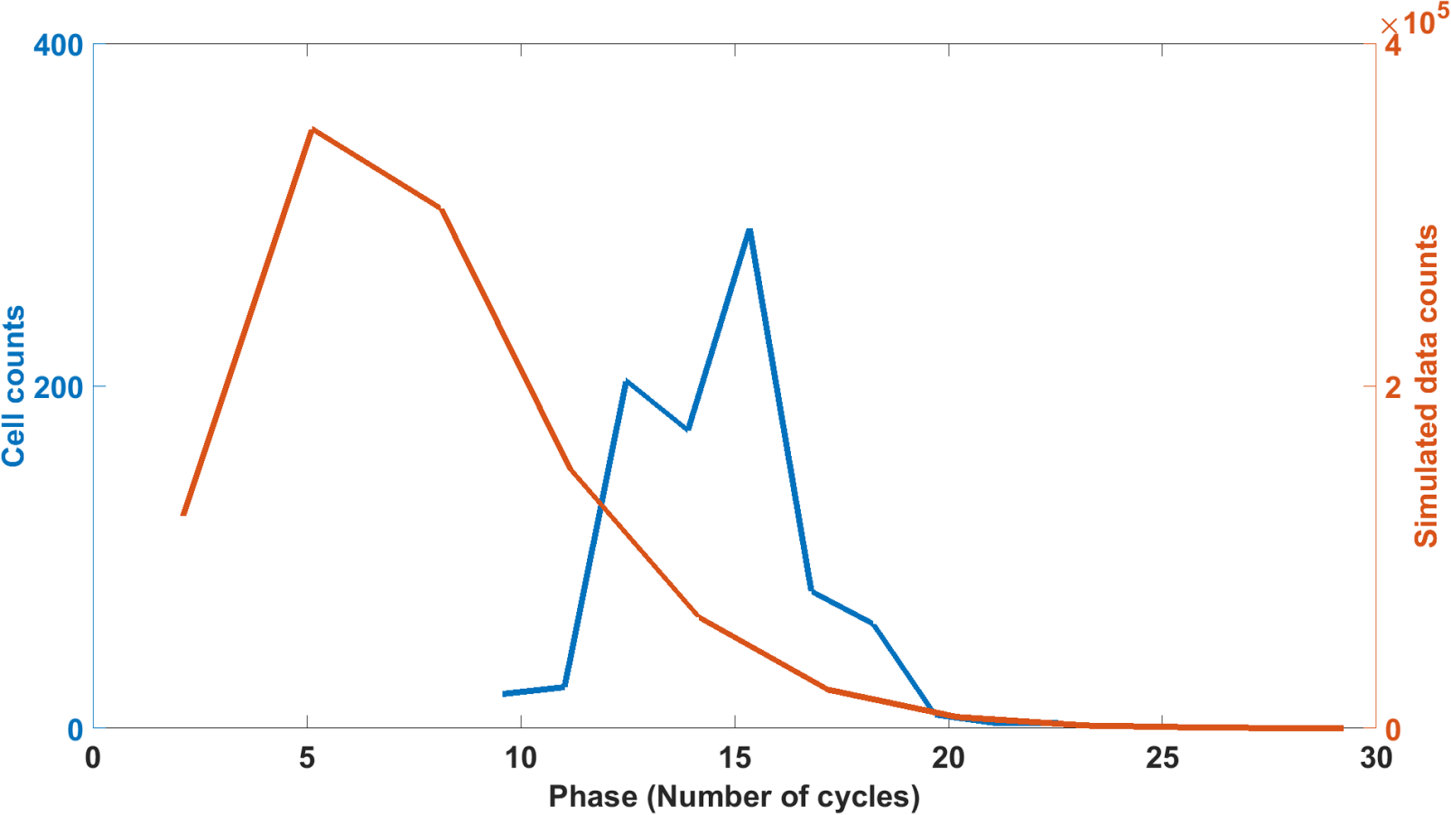
Goodness of fit for the model ensemble is tested with the Hilbert phase for 868 single cells (blue) and Gillespie trajectories (red) under the model with smallest chi-squared statistic in the fitted ensemble (Fig 3). The computation of the Hilbert phase for each trajectory is described previously over a 30 to 115 hour window.[13]. The model histogram is that of the Hilbert phases for 1024 Gillespie trajectories on each of > 1000 models in the best fitting model ensemble (Fig 3).

The histogram of Hilbert phases for single cells and Gillespie trajectories from the model ensemble are compared as a measure of goodness of fit (Fig 7). There are two sources of variation in the Gillespie trajectories, the random variation in phase between Gillespie trajectories of one model and the variation in phase between models in the ensemble of fitted models. The histogram of Hilbert phases in Fig 7 reflects both sources of variation. For each model, 1024 Gillespie trajectories were simulated, and each Gillespie trajectory has a Hilbert Phase. In addition the process was then repeated for over 1000 models in the fitted ensemble (Fig 3) to generate all the values for the model histogram (Fig 7). We see that the histogram for the Gillespie trajectories for over a thousand models in the fitted ensemble, covers the histogram measured on single cells over an 85 hour window. The difference between the two histograms is significant by a Kolmogorov-Smirnov (KS) nonparametric test. We carried out a KS-test of the difference of the two histograms (P < 0.0001) with the maximum difference in cumulative histograms being 0.1747[57].

There are three possible reasons for the discrepancy between phase predicted and phase observed. The systems is experiencing Stochastic Resonance[38–40]. A second possible reason for the discrepancy is cell-to-cell synchronization by quorum sensing. It is unlikely that a quorum sensing mechanism is at work because the cells are physically isolated in droplets in Fig 7[13]. A third reason could be that the Hilbert phase results are dominated by noise fluctuations.

### Is there an intermediate optimum in the oscillatory signal as a function of the stochastic intracellular noise?

**Fig 8:**
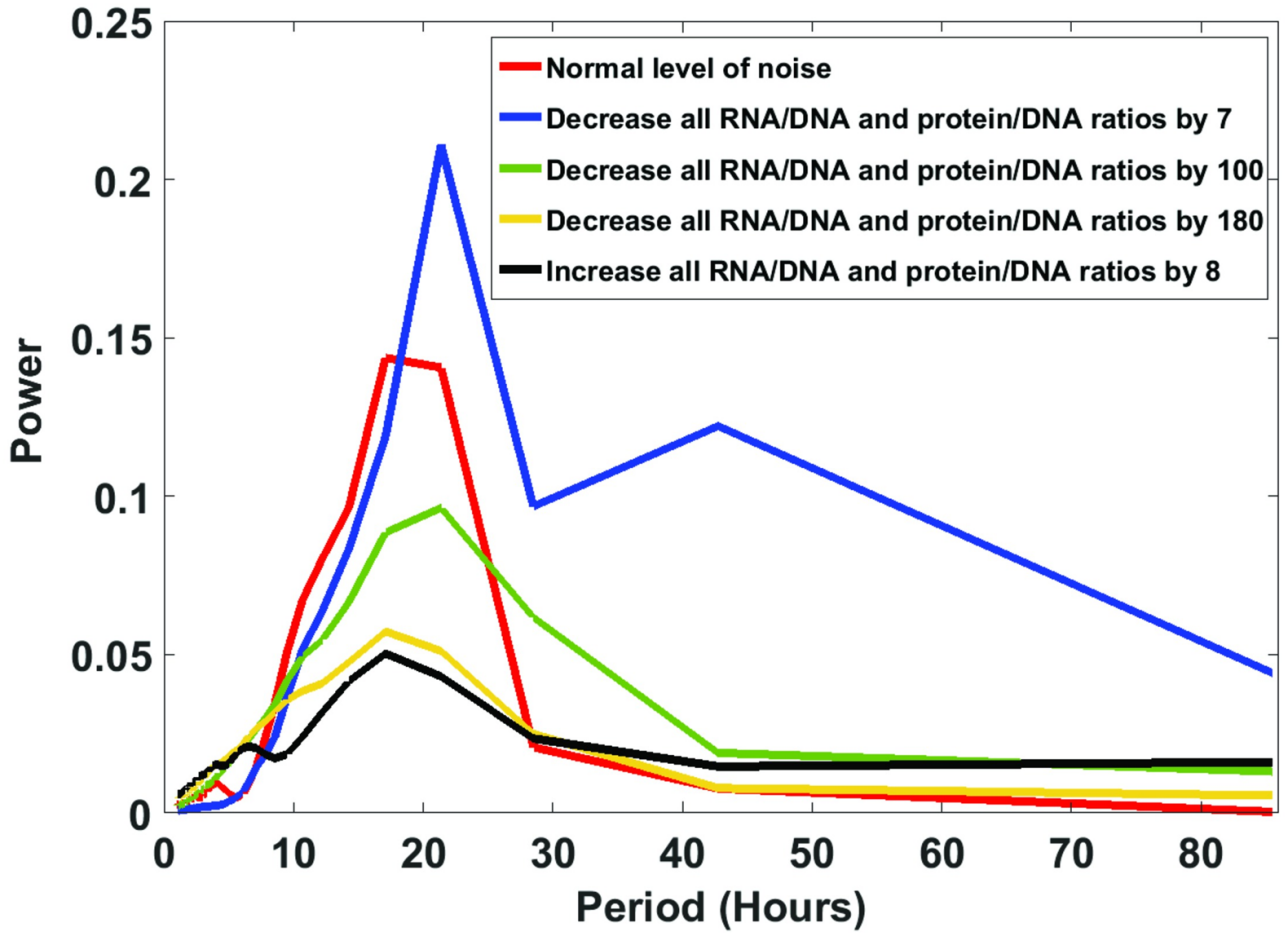
There is a non-monotonic relation between the oscillatory signal strength in the normalized periodogram for the CCG protein species and the stochastic intracellular noise. The red curve is for the best fitting model (Fig 3B), using the observed RNA/DNA and protein/DNA ratios of 128.7 and 412, respectively. The blue, green and yellow curves have a bigger stochastic intracellular noise than the best fitting model, by shrinking the protein/DNA and RNA/DNA ratios 7-, 100- and 180-fold, respectively, relative to the observed values. The black curve has a smaller stochastic intracellular noise by increasing the protein/DNA and RNA/DNA ratios by a factor of 8. The underlying model was determined without bias correction using Eq (1) for 868 single cells.

One possible explanation for the results on goodness of fit may be synchronization through the Stochastic Resonance mechanism acting on isolated single cells. Under this hypothesis there is a non-monotonic relation between peak height (i.e., signal strength) in the periodogram (Fig 3) and the estimated stochastic intracellular noise[38]. Here we examine how the periodogram, which captures the oscillatory signal, varies, as the noise is varied (Fig 8).

One of the clearest examples of the effects of stochastic resonance is in a simple two-dimensional system, in which the polar coordinates (r, *θ*) evolve according to the following dynamical system[39]:

$$\dot{r}\;=\;r(1-r^{2})\;+\epsilon_{1}(t)$$

$$\dot{\theta }\;=\;b-r^{2}\text{cos}\left(2\theta \right)+\;\epsilon_{2}(t)$$

where the *ϵ*-terms are the noise terms. There are two fixed points to this system, and a limit cycle in the deterministic system without the *ϵ*-terms exists for b > 1[39]. What is interesting that in the presence of sufficient noise and b < 1, there is directional flow between the fixed points and hence oscillations. If there is too little noise, the dynamical system cannot escape from the stable fixed points and does not oscillate; likewise, too much noise will also wipe out the oscillations. This model illustrates the hypothesis of stochastic resonance.

Consistent with the Stochastic Resonance hypothesis, too little noise may not allow our dynamical system in Fig 1 to escape stable fixed points; likewise, too much noise may not allow the dynamical system in Fig 1 to settle into a flow between stable fixed points. Yet, if there is the right level of noise, an oscillatory signal may emerge in the periodogram. Here we test this hypothesis in the *N*. *crassa* clock using the single cell data.

What we see in Fig 8 is exactly what we would predict under the Stochastic Resonance hypothesis. As the noise is increased above “normal”, the peak in the periodogram is diminished, and the oscillatory signal is diminished. If the noise is decreased sufficiently from “normal”, the peak in the periodogram is also diminished, and the oscillatory signal is diminished, consistent with the trapping of the real dynamical system in stable fixed points. Only at an intermediate level of noise do we see oscillations in single cells in Fig 8 in the periodogram.

As a final note, in Fig 8 the protein/DNA and RNA/DNA ratios were varied to cause a change in the stochastic intracellular noise (Fig 5), while the reaction propensities were left constant. Some might argue from Eq (3) that the lead deterministic term in the CLE should be kept constant while varying the stochastic intracellular noise. In this way the limiting deterministic dynamics would be kept constant while the noise is varied. In comparing models with the same deterministic dynamics, it was necessary to vary the propensities so that the lead term in the CLE did not change using the rescaling method in the Materials and Methods. The result of this experiment was the same outcome as in Fig 8 with the only change that a much higher RNA/DNA and protein/DNA ratio (~3000) was needed to see the non-monotonic response to stochastic intracellular noise in Fig 8.

## Discussion

In order to describe the stochastic behavior of single cell oscillators, a variety of methodological challenges needed to be surmounted. First and foremost, a scalable fitting method that would work with thousands of trajectories on single cells was needed. Existing methods do not operate on this scale. To overcome this challenge a fast scalable ensemble method for stochastic networks was developed using the periodogram or power spectrum of an average model trajectory to be compared with the average periodogram over single cells. The method is scalable to thousands or tens of thousands of cells on GPUs.

One of the limitations of this approach is that normal Metropolis Hastings ensemble methods[28] are not adequate for stochastic networks. Parallel tempering methods are shown to work well on the stochastic networks examined here (Fig 2). As more complicated alternatives to the model in Fig 1 are considered, surmounting the rate limiting step of generating a Gillespie trajectory may be achieved by other means than through GPUs alone. A promising avenue is generating approximations to the Gillespie trajectory with Quasi Steady-State Approximations (QSSA) to the full stochastic model considered here[58]. The right steady state approximation can help the identifiability of fitting methods for stochastic networks even in simple networks[59].

A second challenge in the fitting process is that there are two sources of error in single cell trajectories, the stochastic intracellular noise and the experimental error[13]. The latter introduces biases into the fitting process. We developed a statistical methodology to remove the experimental error from the fitting process to the periodogram of the model ensemble and thereby achieved a better specification of the model. This new procedure for removing the bias of the experimental error from the periodogram is presented and utilized.

Another challenge of stochastic network identification is characterizing the size of the cell, which sets the level of stochastic intracellular noise, a parameter missing in deterministic models[44]. We developed an empirical approach to identifying the cell size for a stochastic network from the protein/DNA and RNA/DNA ratios for the system under study. We showed that the fitted model was quite robust to variation in these ratios (Fig 6). A final challenge is developing protocols to make single cell measurements[60].

In the study of stochastic periodic phenomena we need both ways to fit such models with large amounts of single cell data as well as ways to test the success of these models. Three statistics provide useful summaries of periodic stochastic networks: period, amplitude, and phase of single cell trajectories. Fortuitously the periodogram is functionally independent of the phase of single cell trajectories[13]. A goodness of fit statistic was then developed using the phase, which included variation in phase across trajectories and the model ensemble.

We then applied these new methodologies to understand the oscillatory behavior of single cells in *N*. *crassa*. We found that parallel tempering was quite successful in identifying a stochastic network to describe the oscillator in single cells. In many cases the rate constants of macroscopic deterministic models based on millions of cells were similar to those in microscopic stochastic models of single cells (Table 2). Yet, there were also some key differences. For example, in macroscopic models the half-life of the *wc-1* mRNA was measured to be quite long and found to be a critical feature in maintaining oscillations[28]. Yet at the single cell level the half-life was estimated to be much shorter. One possible explanation may be that single cells have other mechanisms to produce oscillations than those that operate at the macroscopic scale. For example, single cells experience stochastic intracellular noise that can move cells from one stationary state to another[39], and this behavior may generate oscillations. This phenomenon depends on not having too much or too little stochastic intracellular noise. We show this phenomenon of Stochastic Resonance[38, 61] may play a role in seeing clock-like behavior in single cells (Fig 8). The kinetics of single cells may operate under a set of more relaxed rules for oscillation than those that apply to millions or tens of millions of cells.

Other hypotheses for explaining single cell oscillations need testing[61], such as quorum sensing[62] or cell-cycle gated circadian rhythms[63]. Cell-cycle gating was controlled by choosing a medium inhibiting cell division[13]. The quorum sensing hypothesis would require communication between cells. Yet, the cells examined here were isolated in droplets. In future work cells will be allowed to share the same droplet, and the associations between cells within droplets provide additional statistics to supplement the periodogram to distinguish between quorum sensing and stochastic resonance. Here we have created both an experimental and methodological arrangement to study the physical theory of Stochastic Resonance in living cells. For the first time, we have a window on a new set of dynamics at the single cell level that play by a different set of rules potentially than ensembles of millions of cells.

## Supporting information

S1 Fig Bar charts of the parameters from the macroscopic deterministic model (Table 2 in column 3) in blue and single cell stochastic model (Table 2 in column 6) in orange. Parameters are subdivided into: (A) decay rates; (B) transcription and translation rates; (C) interaction parameters between genes and their products on a log scale; (D) initial conditions for large initial molecular numbers per cell; (E) initial conditions for large initial molecular numbers per cell.
(PDF)

S1 File Fluoresecent data from a recorder downstream of a *ccg-2* promoter from 1591 single cells stored in an excel file. Rows are different times, and columns are different cells. Time points are spaced at half-hour intervals over more than 11 days.
(CSV)

S1 Table Summary features of MCMC runs with parallel tempering for the 868 and 1591 single cell data sets. (DOCX)
