# Supplementary figures and images for: Ensemble methods for stochastic networks with special reference to the biological clock of Neurospora crassa
Source: PLoS One. 2018 May 16;13(5):e0196435. doi: 10.1371/journal.pone.0196435 (PMC5955539; doi:10.1371/journal.pone.0196435)

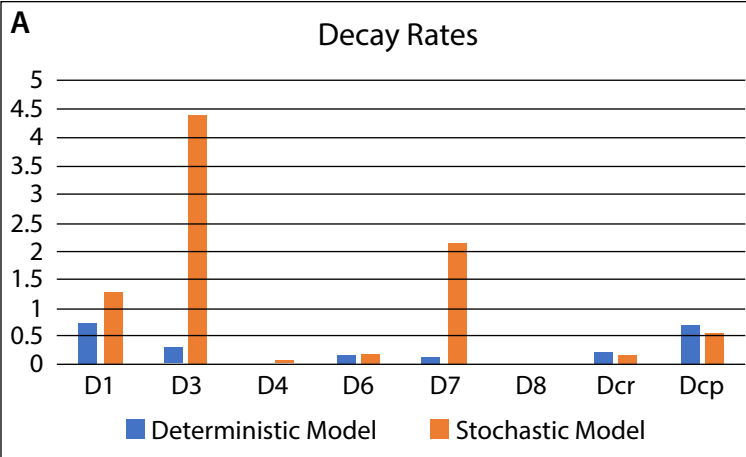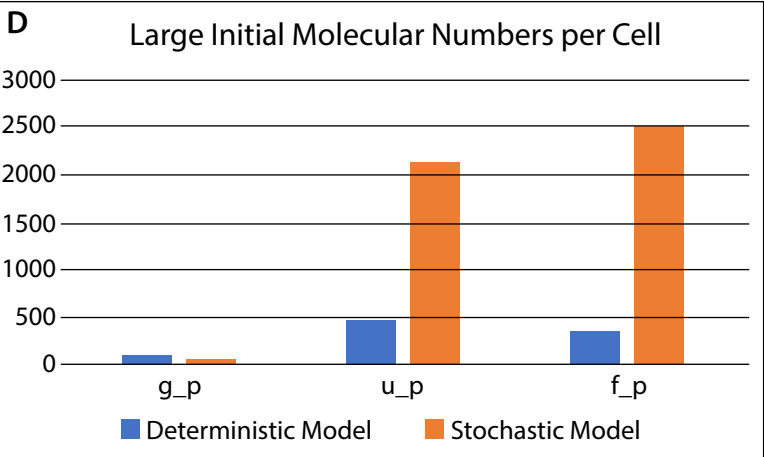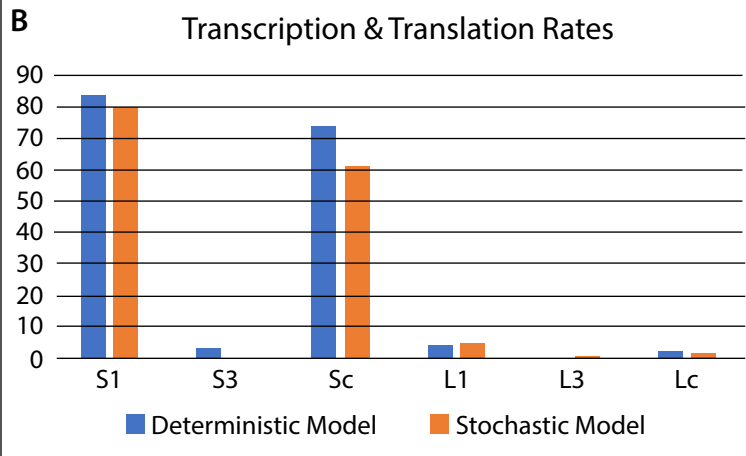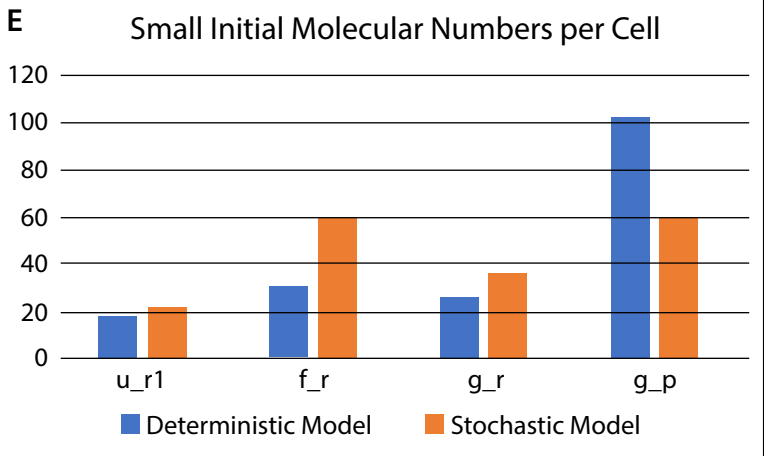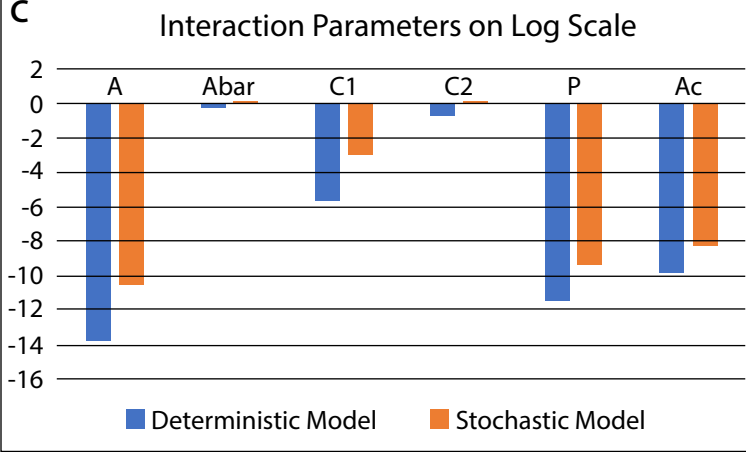

Supplement: S1 Fig — Parameters are subdivided into: (A) decay rates; (B) transcription and translation rates; (C) interaction parameters between genes and their products on a log scale; (D) initial conditions for large initial molecular numbers per cell; (E) initial conditions for large initial molecular numbers per cell. (PDF) [file pone.0196435.s001.pdf]
