# Supplementary material for: Ensemble methods for stochastic networks with special reference to the biological clock of Neurospora crassa
Source: PLoS One. 2018 May 16;13(5):e0196435. doi: 10.1371/journal.pone.0196435 (PMC5955539; doi:10.1371/journal.pone.0196435)
Supplement: S1 Table — (DOCX) [file pone.0196435.s003.docx]

Parallel Tempering Markov Chain Monte Carlo (MC) Features in 868-cell data set

| Parallel Tempering run | Ensemble 1 | Ensemble 2 | Ensemble 3 | Ensemble 4 |
| --- | --- | --- | --- | --- |
| Gillespie trajectory per MC update | 7*1024 | 6*1024 | 6*1024 | 4*1024 |
| Avg. number of $\tau$-steps in a Gillespie trajectory for an MC update | 1490217 | 1854479 | 1926150 | 1312186 |
| Number MC updates for equilibration | 20000 | 20000 | 20000 | 20000 |
| Number of parallel tempering replica exchanges per replica for equilibration | 40000 | 40000 | 40000 | 40000 |
| Number MC updates for accumulation | 37143 | 45595 | 42401 | 54000 |
| Number parallel tempering replica exchanges per replica for accumulation | 68286 | 91190 | 84802 | 108000 |
| Mixing ratio intra-replica/replica exchange updates | 1/2 | 1/2 | 1/2 | 1/2 |
| Number of $\Theta$-vectors included in accumulation MC sample | 1093 | 1342 | 1248 | 1589 |
| Number round trips for equilibration | 125 | 153 | 108 | 164 |
| Number of round trips for accumulation | 59 | 125 | 69 | 67 |
| Avg. time in seconds for simulating Gillespie Trajectories in an MC update | 41.98 | 34.1 | 30.1 | 16.9 |

Parallel Tempering Markov Chain Monte Carlo(MC) Features 1591-cell data set

| Parallel Tempering run | Ensemble 1 | Ensemble 2 | Ensemble 3 | Ensemble 4 |
| --- | --- | --- | --- | --- |
| Gillespie trajectory per MC update | 13*1024 | 14*1024 | 13*1024 | 11*1024 |
| Average number of $\tau$-steps in a Gillespie trajectory for an MC update | 8804667 | 7137363 | 6323311 | 2015506 |
| Number MC updates for equilibration | 3400 | 3400 | 3400 | 3400 |
| Number of parallel tempering replica exchanges per replica for equilibration | 6800 | 6800 | 6800 | 6800 |
| Number MC updates for accumulation | 1341 | 2356 | 4495 | 27100 |
| Number parallel replica exchanges per replica for accumulation | 2682 | 4712 | 8990 | 54200 |
| Mixing ratio intra-replica/replica exchange updates | 1/2 | 1/2 | 1/2 | 1/2 |
| Number of $\Theta$-vectors included in accumulation MC sample | 40 | 70 | 133 | 798 |
| Number round trips in equilibration phase | 18 | 2 | 5 | 8 |
| Number of round trips in accumulation phase | 2 | 2 | 1 | 111 |
| Average time in seconds for simulating Gillespie  Trajectories in an MC update | 369.6 sec | 280.9 | 188.6 | 39.4 |

Notes: MCMC runs have two phases, equilibration and accumulation. The MCMC run’s length was measured in terms of the number of MC updates. In the context of parallel tempering an MC update is equivalent to an intra-replica update. For each MC update, 1024 Gillespie trajectories were performed. In the tables above the MC update was multiplied also by the number of replicas used. To obtain the MC sample in the accumulation phase every 34^th^ $\Theta$-vector was used. The dimension of the parameter vector was 34. A convenient way to measure the mixing in parallel tempering is the number of round trips through the temperature grid that an updated parameter makes.
